# Secondary traumatic stress and compassion satisfaction mediate the association between stress and burnout among Korean hospital nurses: a cross-sectional study

**DOI:** 10.1186/s12912-021-00636-w

**Published:** 2021-06-30

**Authors:** Hyangkyu Lee, Wonhee Baek, Arum Lim, Dajung Lee, Yanghee Pang, Oksoo Kim

**Affiliations:** 1 Department of Nursing, Graduate School of Yonsei Univeristy, Seoul, Republic of Korea; 2 Mo-Im Kim Nursing Research Institute, College of Nursing, Yonsei University, Seoul, Republic of Korea; 3 Department of Nursing, Kyungnam University College of Health Sciences, Changwon, 51767 Republic of Korea; 4 Severance Hospital, Seoul, Republic of Korea; 5 College of Nursing, Ewha Womans University, Seoul, Republic of Korea; 6 Ewha Research Institute of Nursing Science, Seoul, Republic of Korea

**Keywords:** Burnout, Compassion satisfaction, Mediation analysis, Secondary traumatic stress, Stress, The Korean nurses’ health study

## Abstract

**Background:**

Burnout among nurses is a worldwide public health epidemic that adversely affects nurses’ quality of life as well as the patient’s outcomes. The aim of this study was to evaluate the influence of stress on nurses’ burnout and to identify the mediating effects of secondary traumatic stress and compassion satisfaction among clinical nurses in South Korea.

**Methods:**

A quantitative, cross-sectional study evaluated the survey data from 10,305 female registered hospital nurses who participated in the Korea Nurses’ Health Study (KNHS) Module 5. The survey included a demographic questionnaire and the Professional Quality of Life version 5 (ProQOL 5). Bootstrap analyses (using the PROCESS macro) were employed to evaluate the mediating effect between variables.

**Results:**

Stress was significantly associated with burnout and mediated by secondary traumatic stress and compassion satisfaction (β_indirect 1_ = 0.185, Bootstrap confidence interval (BS *CI*) [0.175, 0.194]; β_indirect 2_ = 0.226, BS *CI* [0.212, 0.241], respectively). In addition, the magnitude of the indirect effects of compassion satisfaction was significantly greater than the magnitude of the indirect effects of secondary traumatic stress (β_indirect 1_-β_indirect 2_ = − 0.042, BS *CI* [− 0.058, − 0.026]). The findings of this study indicate that the positive aspect (compassion satisfaction) of work experiences can offset the negative aspects (secondary traumatic stress), consequently reducing burnout level.

**Conclusions:**

Our study findings suggest that a multidimensional approach to assessing nurse burnout and implementation of proper management will improve quality of life for nurses and help maintain positive attitudes and quality of patient care.

## Background

Burnout is a syndrome characterized by three dimensions: emotional exhaustion, depersonalization, and reduced personal accomplishment [1]. People who experience burnout are more likely to leave their jobs; in fact, some resign from their jobs without hesitation. Even if they stay in their jobs, their job performance, efficacy, and job satisfaction are significantly decreased. Moreover, burnout adversely affects physical symptoms, like pain, as well as mental health, including depression and anxiety [2, 3]. Indeed, burnout was recently classified as an occupational phenomenon in the 11th Revision of the International Classification of Diseases (ICD-11) [4] indicating that burnout has emerged as a worldwide health problem in workplaces.

Numerous studies have demonstrated an association between work-related or personal stress and burnout [5–7]. Nurses perform tasks that require professional knowledge and a high level of technical skills; they are also required to cope with patients who have various health needs. Furthermore, nurses experience elevated stress in the course of providing continuous care for patients 24 h a day, as well as contacting and communicating with many medical staff and family members [8–10]. This results in chronic stress build-up, leading to nurses’ burnout [11]. Stress affects the incidence of burnout, and that burnout eventually negatively affects nurses’ general health [12]. In addition, increased burnout is related to lower quality of nursing care, lower patient satisfaction, and higher healthcare-associated infection rates [13].

Nurses experience secondary traumatic stress (STS), defined as negative behavior and emotion driven by fear and work-related trauma, in the course of caring for patients. STS occurs when nurses are traumatized by their work, and is usually associated with a particular event [14]. However, nurses also experience compassion satisfaction (CS) which is a positive emotion that reflects the rewards of caring for others. CS occurs as a result of working with patients and families and experiencing positive emotional rewards such as fulfillment, joy, and hope [15]. As such, professional quality of life (ProQOL) encompass positive and negative aspects; thus, when discussing work-related quality of life of nurses, it is necessary to consider the influential effects or interactive dynamics between burnout, secondary traumatic stress, and compassion satisfaction.

A study conducted in China indicated that, after adjusting for the covariates, longer working shifts were associated with higher STS [16]. Another study conducted in Israel demonstrated that stress and CS were negatively correlated [17]. Moreover, a number of studies reveal that stress is related to or affects STS and CS [18, 19]. In addition, positive (CS) or negative (STS) feelings experienced by nurses may affect burnout [20–22]. In a meta-analysis by Zhang et al. [22] which included 11 studies burnout and STS displayed a strong positive correlation (r = 0.59), and burnout and CS was moderately negatively correlated (r = − 0.446). However, there is a limited number of studies confirming the relationship between stress and STS and CS in nurse burnout [23–25] and it remains unclear what role of STS and CS play in this relationship. Hence, we sought to elucidate how STS and CS function as mediators of burnout.

A high turnover or resignation of nursing staff results in a tremendous nursing shortage [26] and burnout has a major impact on this outcome. According to a report from the National Academies of Science at the end of 2019, 35% of US nurses experience substantial symptoms of burnout [10]. The situation in Korea is more serious, a systematic review of burnout confirmed that Korean nurses had increased burnout compared to nurses in other countries [27]. In addition, the national survey of health workers in Korea reported that physical and psychological burnout was ranked third as the reason for resigning or changing jobs among nursing staff [28]. Although the Korean nurses’ turnover rate is substantially high, no studies have employed national representative sampling and/or factors affecting the burnout of Korean nurses in a clinical setting. Therefore, to reduce burnout among Korean nurses, it is necessary to identify the factors that affect burnout and the relationship between them.

## Methods

This research followed the Strengthening the Reporting of Observational Studies in Epidemiology (STROBE) guideline [29].

### Aim

The purpose of this study was to identify whether stress affects burnout and to confirm whether STS and CS play a mediating role in the relationship between stress and burnout among nurses in Korea. The hypothesis of this study was as follows: Elevated stress is associated with high burnout and the association between stress and burnout is mediated through STS and CS.

### Study design and sample

The Korea Nurses’ Health Study (KNHS) was a prospective cohort study of Korean female registered nurses that examined the effects of occupational, environmental, and lifestyle risk factors on the health of Korean women [30]. The Nurses’ Health Study (NHS) was a cohort study of United States nurses that began in 1976 [31]. The KNHS is a Korean version of the NHS and is based on the study protocol and questions used in the NHS3. The participants of the KNHS were selected from among those who were living in Korea, were between 20 and 45 years of age, and who had at least one year of nursing experience. There were 157,569 women of childbearing age who were registered with the Korean Nurses Association (KNA). The sample size was calculated as 17,431 with a significance level of 0.05 and a permissible error of 1% by random sampling based on the parameters of the population. The target sample size for KNHS was 20,000 female nurses. Module 1 (the baseline survey) was implemented in 2013 and subsequent follow-up surveys were conducted every 6–8 months. Module 5 was conducted in 2016. Nurses were involved in the research through several channels, including social media and print advertising, and surveys were conducted through the KNHS website. Nurses not working in module 5 and nurses not working in hospitals were excluded from the analysis.

### Measurements

#### Demographic characteristics

Eligible registered nurses completed a web-based self-reported questionnaire including data for age, nursing education level, marital status (never married or married), hospital size, department (inpatient, intensive care unit (ICU)/emergency department (ED), operating room, outpatient, management, or others), clinical nursing experience (under 3 years, 3–5 years, 6–10 years or, more than 11 years), work overtime (yes or no), employment (full time or part time), rotational night shift (yes or no) and annual income converted to US dollars per year.

#### Burnout, secondary traumatic stress, and compassion satisfaction

The Professional Quality of Life Scale (ProQOL) version 5 developed by Stamm [14] was used to evaluate the positive and negative aspects of professionals who work to help others. The ProQOL5 is composed of three subscales: STS, CS, and burnout. Each subscale measures separate aspects and cannot be combined [14], has 10 questions rated on a 5-point Likert scale, and has a score range of 10 to 50 points. Scores < 22 points are considered “low”, 23–41 points “moderate”, and > 42 points “high”; the higher the score, the higher the CS, STS, and burnout. ProQOL version 5 developed by Stamm is accessible to the public via web link [32]. The reliability and validity of the ProQOL5 have been validated among Korean nurses [33]. The internal consistency (Cronbach’s α) was previously reported as 0.72 for the STS subscale, 0.89 for the CS subscale, and 0.73 for the burnout subscale [33].

#### Stress

Stress was measured by the perceived stress scale (PSS) developed by Cohen and Williamson [34]. PSS assesses subjective perceptions of stress over the past month. It consists of a total of 10 items rated on a 5-point Likert scale ranging from 4 (fairly frequent) to 0 (none). Scores range from 0 to 40, with higher scores indicating higher levels of stress. Recently, this measure has shown evidence of good psychometric properties in nurse populations [35, 36]. The Cronbach’s α coefficient of the original study was 0.78.

### Statistical analysis

All statistical analyses were performed using R, version 4.4.0 (R Foundation for Statistical Computing) and *p* < 0.05 was considered statistically significant. Descriptive statistics, such as percentages and mean of demographic characteristics were calculated. To identify factors associated with burnout prior to the regression analysis, independent t-tests, ANOVAs, and Bonferroni multiple comparisons were performed according to the characteristics of the variables. To identify the correlation between stress, STS, CS, and burnout, Pearson correlations were performed.

**Fig. 1:**
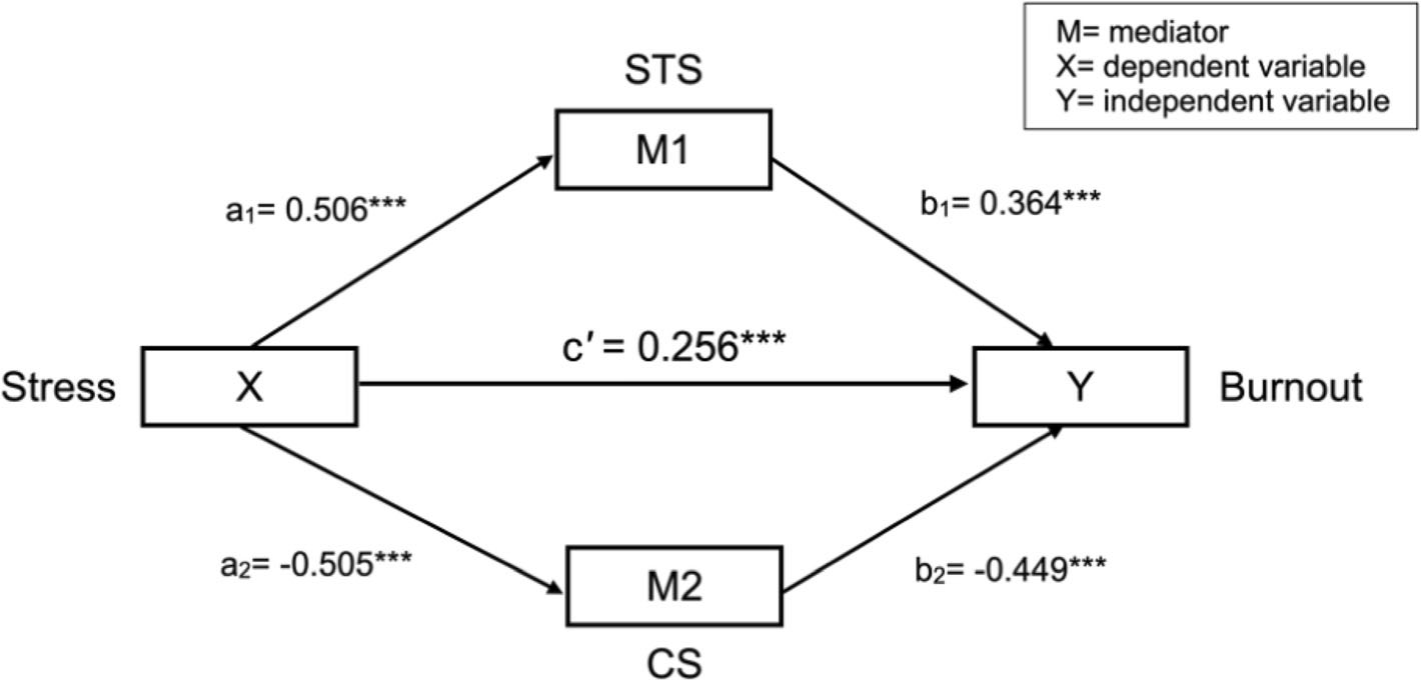
Path diagram for the model. Path coefficients were non-standardized estimates. STS = secondary stress trauma; CS = compassion satisfaction; ***p < .001

The associations between the study variables were analyzed using linear regression analyses and to correct for Type I error due to multiple comparisons, tests were performed at the significance level to which Bonferroni correction was applied. The mediating role of stress and STS, and stress and CS were tested using bootstrap analyses with a PROCESS macro developed by Hayes [37]. In the parallel multiple mediator model, there are three pathways by which the independent variable can be associated with the dependent variable: the direct pathway (c′) leads directly from the independent to the dependent variable, while the indirect pathway ((a_1_ × b_1_) and (a_2_ × b_2_)) incorporates a mediating variable (Fig. 1). First, we tested the indirect effect. After the indirect effect was proven, the direct effect was tested. If a direct effect was identified, it indicated that the relationship between X and Y was partially mediated by M1 and M2 (Fig. 1). The indirect and direct effects were considered statistically significant if the 95% confidence interval of the bootstrap estimate did not include zero. The bootstrap method is preferred in mediation analysis because it uses resampling with replacement and does not require the assumption of normality. Therefore, an accurate inferential test is possible and power is high [37]. All analyses were adjusted for marital status, final education, clinical nursing experience, hospital size, department, overtime work, and rotational night shifts.

## Results

### General demographics and characteristics

In this study, among 11,526 nurses who completed KNHS Module 5, we excluded 1206 who did not work at a hospital and 15 who provided incomplete data. The final sample available for analysis included 10,305 nurses. The demographic and work-related characteristics of nurses are presented in Table 1. This study included 10,305 female nurses with a mean age of 32.8 (SD = 6.06) years (range = 23–51), 59.9% of respondents held a bachelor’s degree. The largest percentage of nurses worked in inpatient departments (40%).

**Table 1:** Burnout according to Korean nurses’ demographics and characteristics (*N* = 10,305)

			Burnout			
	*N*	%	*Mean*	*SD*	*F* or *t*	*p*
Education					64.89	<.001
Diploma (3-yr course) ^a^	2706	26.2	27.2	5.3		b,c < a ^$^
Bachelor’s ^b^	6168	59.9	26.9	5.2		c < b ^$^
Master’s or higher ^c^	1431	13.9	25.3	5.1		
Marital status					21.13	<.001
Never married	4873	47.3	27.9	5.3		
Married	5432	52.7	25.7	5.0		
Hospital size (number of beds)					6.83	<.001
30–299 ^a^	2390	23.2	26.4	5.2		a < b ^$^
300–599	2187	21.2	26.7	5.3		
600–999 ^b^	3698	35.9	27.0	5.2		
1000 or more	2030	19.7	26.8	5.3		
Department					70.75	<.001
Inpatient ^a^	4123	40.0	27.5	5.2		c,d,e,f < a ^$^
ICU/ED ^b^	1629	15.8	27.3	5.1		c,d,e,f < b ^$^
Operating room ^c^	996	9.7	26.5	5.0		e,f < c ^$^
Outpatient ^d^	1723	16.7	26.2	5.3		e < d ^$^
Management ^e^	1046	10.2	24.7	5.0		e < f ^$^
Others ^f^	788	7.6	25.6	5.2		
Clinical nursing experience (yrs)					116.50	<.001
under 3 ^a^	460	4.5	28.3	5.3		c,d < a ^$^
3–5 ^b^	1724	16.7	27.9	5.1		c,d < b ^$^
6–10 ^c^	3714	36.0	27.3	5.3		d < c ^$^
11 or more ^d^	4407	42.8	25.7	5.0		
Work overtime					−11.17	<.001
No	1196	11.6	25.2	5.2		
Yes	9109	88.4	27.0	5.2		
Employment					−1.36	.174
Full-time	9611	93.3	26.7	5.2		
Part-time	694	6.7	27.0	5.4		
Rotational night shift					−16.66	<.001
No	4538	44.0	25.8	5.1		
Yes	5767	56.0	27.5	5.2		
Income (1000 USD)/year					10.58	<.001
36.9 or less	7543	73.2	27.1	5.2		
37 or more	2762	26.8	25.8	5.2		

*ED* emergency department, *h* hours, *ICU* intensive care unit, *SD* standard deviation, *yrs*. years, ^$^ = Bonferroni post hoc test

If there were a difference in the mean value for demographics and characteristics, a post-hoc analysis was performed using the Bonferroni method. For categories with significant differences, a, b, c, d, and e were given as superscript letters and relative sizes were indicated

### Burnout according to general characteristics

Cronbach’s α coefficient of burnout in our study was 0.73. Burnout levels, according to general characteristics of the participants, are presented in Table 1. In terms of demographic characteristics, there were significant differences in education level (*F* = 64.89, *df* = 2, *p* < .001) and marital status (*t* = 21.13, *p* < .001). The group of nurses with diploma degrees experienced higher burnout than nurses with a bachelor’s, or master’s or higher degree. Never-married nurses experienced higher burnout, with a score of 27.9 (SD = 5.0).

For work-related characteristics, nurses working in a hospital with 600–999 beds had the highest burnout score of 27.0 (SD = 5.2) compared to nurses working in a hospital with 30–299 beds (*F* = 6.83, *df* = 3, *p* < .001) and those working in inpatient or ICU/ED departments had the highest scores of 27.5 (SD = 5.2), and 27.3 (SD = 5.1), respectively (*F* = 70.75, *df* = 5, *p* < .001). In addition, for nurses with less than 6 years of working experience (*F* = 116.5, *df* = 3, *p* < .001), overtime work (*t* = − 11.17, *p* < .001), rotational night shifts (*t* = − 16.66, *p* < .001), and lower annual income (*t* = 10.58, *p* < .001), burnout scores were relatively higher.

### Burnout level and relationship among stress, secondary traumatic stress, and compassion satisfaction

Cronbach’s α coefficients for stress, STS, and CS in our study were 0.7, 0.72, and 0.89, respectively. The mean stress score of 10,305 nurses was 17.7 (SD = 4.5), the mean STS score was 24.2 (SD = 5.8), the mean CS score was 30.9 (SD = 7.1), and the mean burnout score was 26.7 (SD = 5.3). There was a positive relationship between stress and burnout as well as stress and STS (*r* = 0.60, *p* < .001; *r* = 0.40, *p* < .001, respectively). Stress and CS were negatively correlated (*r* = − 0.35, *p* < .001). Burnout and STS were positively correlated (*r* = 0.47, *p* < .001) while burnout and CS were negatively correlated (*r* = − 0.67, *p* < .001) (Table 2).

**Table 2:** Level and relation among stress, secondary traumatic stress, compassion satisfaction, and burnout among Korean nurses (N = 10,305)

			Pearson correlation coefficients			
Variable	*Mean*	*SD*	Stress	STS	CS	Burnout
Stress	17.7	4.5	1.00			
Secondary traumatic stress	24.2	5.8	0.40	1.00		
Compassion satisfaction	30.9	7.1	−0.35	0.04	1.00	
Burnout	26.7	5.3	0.60	0.47	−0.67	1.00

*SD* standard deviation, *STS* secondary traumatic stress, *CS* compassion satisfaction

### Multiple linear regression analysis

Descriptive statistics for stress, STS, and CS for the multiple linear regression analysis are shown in Table 3. We performed a multiple linear regression using stress as the predictor variable for STS, CS, and burnout. The adjusted R squares for these regression models were 19, 20, and 73%, respectively. Factors affecting burnout included stress, STS, CS, marital status, hospital size, work overtime, and a higher stress level was associated with a higher burnout level (β = 0.256, *p* < .001). Factors affecting STS included marital status, hospital size, department, work overtime, and a higher stress level was associated with higher STS (β = 0.506, *p* < .001). Factors affecting CS included marital status, final education, department, clinical nursing experience, and a higher stress level was associated with lower CS levels (β = − 0.505, *p* < .001).

**Table 3:** Model estimates for stress, secondary traumatic stress, and compassion satisfaction by multiple linear regression analysis among Korean nurses (N = 10,305)

Variable	Consequent								
	M1: STS			M2: CS			Y: Burnout		
Antecedent	Coef	*s.e*	*p*	Coef	*s.e*	*p*	Coef	*s.e*	*p*
Stress	0.506	0.012	<.001	−0.505	0.014	<.001	0.256	0.007	<.001
*Mediator*									
Mediator 1: STS	–	–	–	–	–	–	0.364	0.005	<.001
Mediator 2: CS	–	–	–	–	–	–	−0.449	0.004	<.001
*Covariates*									
Marital									
Married	Ref(0)			Ref(0)			Ref(0)		
Never married	−0.471	0.121	<.001	−1.785	0.147	<.001	0.412	0.063	<.001
Education									
Over master	Ref(0)			Ref(0)			Ref(0)		
Diploma	−0.427	0.192	0.026	−1.423	0.234	<.001	−0.017	0.100	0.865
Bachelor	−0.206	0.166	0.216	−1.126	0.203	<.001	−0.069	0.087	0.428
Hospital size									
30–299	Ref(0)			Ref(0)					
300–599	0.295	0.157	0.060	−0.074	0.192	0.699	0.204	0.082	0.013
600–999	0.517	0.146	<.001	0.058	0.178	0.746	0.313	0.076	<.001
1000 or more	0.445	0.166	0.007	0.039	0.203	0.847	0.219	0.087	0.012
Department									
Inpatient	Ref(0)			Ref(0)			Ref(0)		
ICU/ED	0.028	0.154	0.858	0.052	0.188	0.006	−0.065	0.080	0.417
Operating R.	−1.412	0.188	<.001	−0.219	0.223	0.341	0.009	0.098	0.928
Outpatient	−1.118	0.181	<.001	0.040	0.221	0.856	−0.115	0.094	0.223
Management	−1.506	0.216	<.001	1.860	0.264	<.001	0.122	0.113	0.282
Others	−1.080	0.221	<.001	0.991	0.270	<.001	−0.116	0.116	0.318
Clinical nursing experience									
Under 3 yr	Ref(0)			Ref(0)			Ref(0)		
3-5 yr	−0.548	0.275	0.046	0.200	0.336	0.550	−0.330	0.143	0.021
6-10 yr	−0.133	0.260	0.609	0.718	0.318	0.024	−0.182	0.136	0.181
11 or more	0.097	0.271	0.722	2.336	0.331	<.001	0.048	0.142	0.736
Work overtime									
No	Ref(0)			Ref(0)			Ref(0)		
Yes	1.107	0.166	<.001	0.247	0.203	0.224	0.416	0.087	<.001
Rotational night shift									
No	Ref(0)			Ref(0)			Ref(0)		
Yes	0.348	0.143	0.015	−0.017	0.174	0.924	−0.065	0.745	0.381
*R*^*2*^	0.19			0.20			0.73		
Adjusted R^2^	0.19			0.20			0.73		
Residual *SE*	5.21 (*df* = 10,287)			6.36 (*df* = 10,287)			2.72 (*df* = 10,285)		
*F*	139.2 (*p* < .001)			153.8 (*p* < .001)			1482 (*p* < .001)		

*STS* secondary trauma stress, *CS* compassion satisfaction, *Y* dependent, *Coef* coefficient, *s.e* or *SE* standard error, *ICU* intensive care unit, *ED* Emergency department, *Operating R* Operating room, *df* degrees of freedom

### Testing for the mediator

Table 4 presents the results of the bootstrap analyses for the mediation analysis. STS and CS had indirect effect between stress and burnout (β_indirect 1_ = 0.185, Bootstrap confidence interval (BS *CI*) [0.175, 0.194]; β_indirect 2_ = 0.226, BS *CI* [0.212, 0.241], respectively). Stress had a direct effect of burnout (β_direct c′_ = 0.256, BS *CI* [0.240,0.272]) (Fig. 1). Therefore, STS and CS partially mediated the relationship between stress and burnout. In addition, the indirect effect of stress on burnout with CS as the mediator is greater than the indirect effect of stress on burnout with STS as the mediator (β_indirect 1_-β_indirect 2_ = − 0.042, BS *CI* [− 0.058, − 0.026]). The proportion of mediating effects with total effect was 61% (BS *CI* [0.596,0.636]).

**Table 4:** Mediation analyses of secondary traumatic stress and compassion satisfaction in the association between stress and burnout

Effect	Equation	Estimate	95% Bootstrap *CI*
Indirect 1	(a_1_) * (b_1_)	0.185	0.175 to 0.194
Indirect 2	(a_2_) * (b_2_)	0.226	0.212 to 0.241
Direct	c′	0.256	0.240 to 0.272
Contrast	Indirect 1 - Indirect 2	−0.042	−0.058 to − 0.026
Indirect	Indirect 1 + Indirect 2	0.411	0.395 to 0.429
Total	Indirect + Direct	0.667	0.648 to 0.690
Prop.mediated	Indirect / Total	0.616	0.596 to 0.636

*CI* confidence interval, *Prop.mediated* proportion of indirect effect to total effect

## Discussion

In this study, we evaluated the influence of stress on burnout among Korean nurses and examined the mediating effects of STS and CS. The results from the mediation model indicated that stress had not only a direct effect on burnout among Korean nurses but also an indirect effect on burnout via STS and CS. Additionally, the magnitude of the indirect effects of CS was significantly greater than STS.

Based on a nationwide representative sample, the mean burnout score of Korean nurses was 26.7 (5.2) indicating moderate burnout. In previous studies that used the same measurement tool, the mean burnout score for American nurses was 23.66–25.63 which was lower than for nurses in Korea [23–25], while the mean burnout score of Chinese nurses was 26, similar to nurses in Korea [38]. The prevalence rate of overtime work in Korean nurses was 88%, which was considerably higher than the rates observed in China (55%) [39] and Europe (27%) [40]. Moreover, the number of patients per nurse was higher than in Thailand, China, the US, and European countries; a higher nurse to patient ratio (1:12.3) is associated with lower quality care and poor patient safety [41]. In fact, the RN-to-population was 3.5:1000, which is less than half of the mean (7.2:1000) of the Organization for Economic Cooperation and Development (OECD) countries [28].

Our study results revealed a strong positive correlation between stress and burnout, consistent with previous research. In particular, work-related stress is considered a major concern, because burnout symptoms are associated with stress due to job demands and lack of organizational support [6, 7]. Although nurses primarily treat patients’ illnesses and enhance their well-being, they are also required to assist with patients’ circumstances such as family dynamics and social support systems. In this process, nurses who provide 24-h care face difficulty with additional tasks, such as handling unexpected system problems or role conflicts with other medical staff [9]. Conflicts between the patient’s circumstance, institutional system/support, and professional responsibilities of nurses often result in increased overtime work and burnout [8, 27, 41].

On the other hand, Khamisa et al. [5] reported that personal stress rather than work-related stress was a better predictor of burnout and general health. Indeed, it has been reported that when there was a problem with their family, nurses were less able to concentrate on work, which increased burnout [42]. However, the results of our regression analysis revealed that married nurses had lower levels of burnout after accounting for other variables (Table 3). This result supports previous findings that work-related stress or compassion fatigue were alleviated by supportive networks from family and community [43, 44]. There are also gender effects on the prevalence of burnout. Most Korean nurses (95.2%) are female [28] and are responsible not only for work but also for family obligations such as childcare at home. Consequently, they may have to endure stressful situations both inside and outside the workplace [45]. Therefore, we should consider family-work conflict (e.g., how personal stress affects burnout) or how much job stress is buffered by personal situations when individuals perceive situations as stressful. However, since this study is a cross-sectional study, it is difficult to be certain whether perceived stress affects burnout or whether burnout affects perceived stress. Therefore, further large-scale longitudinal study is needed to determine the effect on burnout according to stress.

Taken together, the results of these studies suggest that stress assessment and management are an essential approach to prevent burnout. A recent meta-analysis supported the notion that stress management is one of the major effective interventions to prevent and reduce burnout of physicians [46]. However, it is also necessary to consider the relationship between burnout and stress as a whole given the difficulty with categorizing stress into uniquely “job” or “individual” dimensions [44]. To address nurses’ stress management it is necessary to develop a comprehensive plan that encompasses several characteristics, rather than dividing stress into dimensions and presenting partial solutions.

In this study, we confirmed that STS has an indirect effect on the relationship between stress and burnout. Higher stress levels resulted in higher burnout levels and the additional STS further increased burnout. This finding is consistent with those of a previous study in which nurses who had insufficient time to care for patients due to workload experienced high STS [19]. STS progresses rapidly [14] while burnout progresses gradually due to high workload or an unsupportive work environment [27]. Because STS can be prevented and ameliorated [47], medical institutions need to address STS appropriately and take early, preventative measures to ensure that burnout is not exacerbated.

We also confirmed that CS has a partial mediating effect on the relationship between stress and burnout. This was consistent with the results of a previous study investigating the negative correlation between burnout and CS [20]. CS is a positive outcome of working as a nurse, however, its effects are reduced when experiencing significant stressful situations and, consequently, burnout will occur. Conversely, even if there is a stressful situation, a nurse experiencing CS can counterbalance the relationship between stress and burnout. In particular, the indirect effect of CS was greater than the indirect effect by STS (Table 4) resulting in reducing burnout among nurses. Moreover, high empathy reduces a nurse’s burnout [38], which can be interpreted as an additional positive effect experienced by nurses who experience a sense of reward from helping others, even in difficult situations. Therefore, it would be effective to establish a management strategy to reduce the nurses’ burnout in a way that reduces stress and increases CS. Chen et al. (2018) reported that CS was reinforced by workplace support such as regular debriefing with managers and priests for nurse staff, and CS was associated with personality traits (conscientiousness, affability and emotional stability). In addition, an emotional regulation training program that includes psychoeducation, progressive muscle relaxation, and nonjudgmental awareness has been demonstrated to increase CS [48]. Therefore, an improved organizational approach that encourages a dynamic environment, such as group support or coaching, could help nurses engage with CS.

### Limitations

There are several limitations to this study. First, psychological characteristics are also influential factors that affect burnout [27], but these were not included in the survey. Second, the hospital or manager’s support and relationships with colleagues could not be investigated. Third, PSS is a tool to measure how to perceive stress in stress-related processing, it could not be clearly determined whether the PSS was the appropriate scale for assessing occupational or personal related stress, or if it measured a combination of both. Fourth, the KNHS is a prospective cohort study of female nurses focusing on the effects of occupational, environmental, and lifestyle risk factors on the health of Korean women [30]; thus, male nurses are not represented in the analysis. Although the proportion of male nurses in Korea is extremely low (4.8%) [28] their burnout also needs to be addressed. Fifth, this is a cross-sectional and secondary analysis study, so the results have limited use for making conclusions about causal relationships. These limitations should be addressed in further studies to confirm factors that influence nurses’ burnout.

## Conclusion

This study was the first investigation of the relationship between stress, STS, CS, and burnout among Korean nurses using a nationwide representative sample. We observed that burnout was associated with nurses’ stress. Comprehensive identification of the causes increasing personal and work-related stress and appropriate interventions will help to reduce nurses’ burnout. We found that STS and CS may exert partial mediating effects on the relationship between stress and burnout in this study, therefore, a multidimensional approach, including reduction of STS and promoting CS within a stress-reducing intervention program will be more effective.

## Data Availability

The datasets are not publicly accessible and freely available since the use and analysis of the pooled data and the publication of any research findings and study results out of it are restricted by contract with the Korea Centers for Disease Control and Prevention (KCDC). The KCDC is planning on opening this data to the public in the future.

## Abbreviations

CS: Compassion Satisfaction
ER: Emergency Room
ICD-11: 11th Revision of the International Classification of Diseases
ICU: Intensive Care Unit
KNA: Korean Nurses Association
NHS: Nurses’ Health Study
KNHS: Korea Nurses’ Health Study (KNHS)
OECD: Organization for Economic Cooperation and Development
ProQOL 5: Professional Quality of Life Version 5
PSS: Perceived Stress Scale
STROBE: Strengthening the Reporting of Observational Studies in Epidemiology
STS: Secondary Traumatic Stress
